# Cardiac output‐guided hemodynamic therapy for adult living donor kidney transplantation in children under 20 kg: A pilot study

**DOI:** 10.1111/pan.13705

**Published:** 2019-08-04

**Authors:** Marieke Voet, Anneliese Nusmeier, Jos Lerou, Josianne Luijten, Marlies Cornelissen, Joris Lemson

**Affiliations:** ^1^ Department of Anesthesiology, Pain and Palliative Medicine Radboud university medical center Nijmegen The Netherlands; ^2^ Department of Intensive Care Medicine Radboud university medical center Nijmegen The Netherlands; ^3^ Department of Pediatric Nephrology Radboud university medical center, Amalia Children’s Hospital Nijmegen The Netherlands

**Keywords:** anesthesia, cardiac output, donor–recipient size mismatch, hemodynamic monitoring, pediatric, renal transplantation

## Abstract

**Background:**

A living‐donor (adult) kidney transplantation in young children requires an increased cardiac output to maintain adequate perfusion of the relatively large kidney. To achieve this, protocols commonly advise liberal fluid administration guided by high target central venous pressure. Such therapy may lead to good renal outcomes, but the risk of tissue edema is substantial.

**Aims:**

We aimed to evaluate the safety and feasibility of the transpulmonary thermodilution technique to measure cardiac output in pediatric recipients. The second aim was to evaluate whether a cardiac output‐guided hemodynamic therapy algorithm could induce less liberal fluid administration, while preserving good renal results and achieving increased target cardiac output and blood pressure.

**Methods:**

In twelve consecutive recipients, cardiac output was measured with transpulmonary thermodilution (PiCCO device, Pulsion). The algorithm steered administration of fluids, norepinephrine and dobutamine. Hemodynamic values were obtained before, during and after transplantation. Results are given as mean (SD) [minimum‐maximum].

**Results:**

Age and weight of recipients was 3.2 (0.97) [1.6‐4.9] yr and 14.1 (2.4) [10.4‐18] kg, respectively. No complications related to cardiac output monitoring occurred. After transplantation, cardiac index increased with 31% (95% CI = 15%‐48%). Extravascular lung water and central venous pressure did not change. Fluids given decreased from 158 [124‐191] mL kg^−1^ in the first 2 patients to 80 (18) [44‐106] mL kg^−1^ in the last 10 patients. The latter amount was 23 mL kg^−1^ less (95% CI = 6‐40 mL kg^−1^) than in one recent study, but similar to that in another. After reperfusion, all patients received norepinephrine (maximum dose 0.45 (0.3) [0.1‐0.9] mcg kg^−1^ min^−1^). Patient and graft survivals were 100% with excellent kidney function at 6 months post‐transplantation.

**Conclusion:**

Transpulmonary thermodilution‐cardiac output monitoring appeared to be safe and feasible. Using the cardiac output‐guided algorithm led to excellent renal results with a trend toward less fluids in favor of norepinephrine.

**What is already known**

- Cardiac output increases after adult donor kidney transplantation in small children.
- Perioperative hemodynamic therapy is currently based on high target central venous pressures leading to liberal fluid administration with the risk of tissue edema.

**What this article adds**

- The transpulmonary thermodilution technique for monitoring cardiac output is feasible and safe in living donor kidney transplantation in children under 20 kg.
- Cardiac output‐guided hemodynamic therapy seems to lead to less liberal intra‐operative administration of fluids in favor of norepinephrine while achieving good renal outcomes.

## 1 INTRODUCTION

Renal transplantation is the therapy of choice for children with end‐stage renal disease. Living‐donor kidney transplantation (LDKT) has benefits including less acute rejection, better catch‐up growth and higher quality of life.^1^ Living‐donor kidney transplantation in children implies an adult donor because children are not considered as living donors for ethical reasons. In small children, this leads to a large donor‐recipient size mismatch. The normal blood flow to one adult kidney is approximately 0.5 L min^−1^ or 10% of the cardiac output (CO), whereas the CO of a 1‐year‐old child is approximately 2.5 L min^−1^.^2^ Therefore, kidney transplantation in a young child implies large hemodynamic changes to meet the “adult” flow and pressure requirements of the donor kidney. These changes are scarcely quantified in the literature. Increased aortic blood flow and CO have been reported, but the reported measuring techniques are ill‐suited for routine use during surgery.^3^, ^4^

It is uncertain how to guarantee sufficient blood flow through the transplanted kidney, as evidence‐based guidelines do not exist. Hemodynamic therapy protocols commonly advise liberal fluid administration guided by high target central venous pressure (CVP) and arterial blood pressure (ABP).^5^, ^6^ However, ABP and CVP are known to poorly reflect CO and organ blood flow or predict fluid responsiveness.^7^ Therefore, such therapy may lead to good renal outcomes, but the risk of fluid overload is substantial. This is illustrated by the high incidence of pulmonary edema, prolonged ventilator support, and renal replacement therapy in these patients.^5^, ^6^

In 2012, our center started a special program for LDKT in children under the age of four. Designing the protocol for perioperative hemodynamic support, we aimed at a physiology‐based approach. The high target CVP advocated in literature was abandoned, aiming for less liberal fluid administration. This accords with many reports questioning the use of CVP as a reliable monitor of fluid status in adults.^8^ Based upon our experience with advanced hemodynamic monitoring in children, we chose the transpulmonary thermodilution (TPTD) technique to measure CO as this technique has been validated and is designated as the gold standard in children.^9^, ^10^

As this is the first report on TPTD‐CO monitoring during LDKT with large donor‐recipient size mismatch, our first goal was to evaluate the safety and feasibility of the technique and analyze the hemodynamic changes. The second goal was to evaluate whether this CO‐guided hemodynamic therapy algorithm induced a reduction in intraoperative fluid administration compared with other reports, while achieving increased target CO and ABP with good renal outcome.

## 2 MATERIALS AND METHODS

### 2.1 Study design

**Figure 1:**
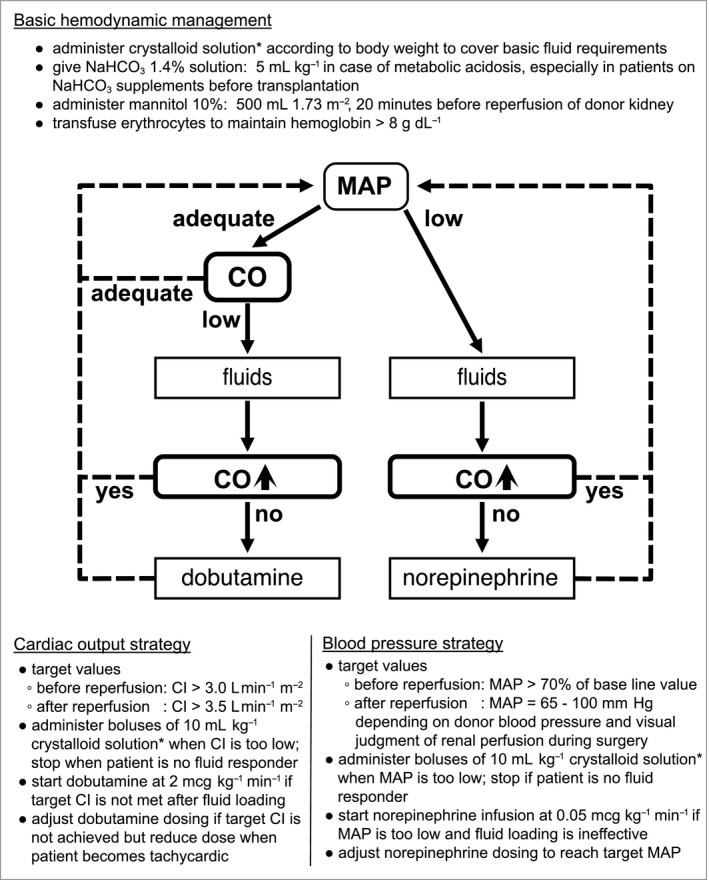
Algorithm of perioperative hemodynamic therapy in pediatric kidney transplantation guided by blood pressure and cardiac output measurements. NaHCO_3_, sodium bicarbonate; MAP, Mean Arterial Pressure; CO, cardiac output; CI, Cardiac Index. Fluid responsiveness is defined as an increase in CO (or stroke volume) of >10%. *Consider using balanced solution (like lactated Ringers' solution) to prevent hyperchloremic acidosis

All children with a bodyweight below 20 kg who underwent a LDKT between the start of the program in October 2012 and September 2016 were consecutively included in this prospective observational study. Two anesthesiologists dedicated to the program alternately provided the anesthetic care using a standardized anesthesia protocol and a hemodynamic therapy algorithm (Figure 1).

### 2.2 Intraoperative management

All patients underwent extensive pre‐operative screening, including cardiac evaluation. The donor nephrectomies were laparoscopic procedures. Donor kidneys were inserted right‐sided intra‐abdominally through a transverse abdominal incision with vascular anastomoses on the aorta and inferior caval vein. All recipients were treated by the TWIST immunosuppressive protocol.^11^

Anesthesia was induced with sevoflurane by face mask or intravenous thiopental (3‐5 mg kg^−1^). After administration of intravenous rocuronium (0.5‐1 mg kg^−1^) an endotracheal tube was placed and positive pressure ventilation started with a tidal volume of 6‐8 mL kg^−1^ and positive end‐expiratory pressure of 4‐6 cm H_2_O. Anesthesia was maintained with sevoflurane (end‐tidal concentration 2.0%‐2.2%) in oxygen/air and intravenous sufentanil. Target end‐expiratory partial pressure for CO_2_ was 33‐38 mm Hg. Cefuroxim was given before surgical incision as a prophylactic antibiotic. Mannitol 10% (0.5 L 1.73 m^−2^) was administered 10 minutes before reperfusion of the donor kidney. No diuretics were given.

### 2.3 Hemodynamic instrumentation

A 22 G and a 4 Fr catheter were placed in a radial artery and the right internal jugular vein, respectively. When the dialysis catheter was already in place, this was used as the central venous catheter. Heart rate (HR), ABP, and CVP were continuously monitored (Philips data monitor). A thermistor‐tipped PiCCO‐catheter (3 Fr, 7 cm) was inserted (ultrasound‐guided) in the left femoral artery to measure TPTD‐CO. This site was chosen as it was as far away as possible from the vascular anastomosis with all transplantations right sided. The catheter was connected to the PiCCO2 device. The device was connected to a laptop computer with PiCCOwin‐software for storage of hemodynamic measurements and curves (Pulsion). The curves were reviewed for quality assessments to assure that they were technically correct.

### 2.4 Data collected

Patients' demographic data, pretransplantation (co‐)morbidities, estimated glomerular filtration rate (eGFR), presence of dialysis, and recipient‐donor weight ratio were recorded. Also, duration of anesthesia, blood loss, vasoactive drug and fluid administration, time to first diuresis, time to creatinine nadir, time to PiCCO‐catheter removal, duration of hospital stay, complications, and eGFR 6 months after transplantation were recorded.

TPTD‐CO measurements were performed at time points left to the discretion of the attending physician. However, three measurements were done at fixed moments in time and were used in our study. All measurements were done in a hemodynamically stable period. Stable conditions were defined as variance in hemodynamic variables of <10% and no change in fluids or vasopressor administration in the last 15 minutes. The first measurement was performed after induction of anesthesia, before surgical incision (t0), the second during surgery after reperfusion of the donor kidney (t1), and the third in the ICU just before removal of the PiCCO catheter (t2). The PiCCO catheter was removed when vasoactive medication was no longer necessary to optimize hemodynamics. At t0, t1, and t2 hemodynamic variables were recorded. CO was measured as the mean of three consecutive measurements with central venous injections of 10 mL iced saline 0.9%. To prevent calculation bias by the PiCCO software, only the TPTD measurements with absolute values of CO and extravascular lung water were used. Cardiac output was divided by the mean HR obtained during TPTD measurements to calculate stroke volume. Cardiac output and stroke volume were adjusted for body surface area, thus yielding CI, expressed as L min^−1^ m^−2^ and stroke volume index (SVI), expressed as mL m^−2^. Extravascular lung water was adjusted for body weight, yielding extravascular lung water index (EVLWI) expressed as mL kg^−1^. Global end diastolic volume was not used and not studied because of lack of validation in children. EVLWI was not used to guide therapy but was studied with regard to individual changes as a marker of pulmonary edema.

### 2.5 Hemodynamic therapy algorithm

Basic fluid requirements were calculated according to the 4‐2‐1‐rule of Holliday and Segar. Intravenous fluids, inotropic and vasopressor support were titrated according to the algorithm shown in Figure 1 to reach target CO and mean arterial pressure (MAP). Crystalloids were chosen as primary fluids as semisynthetic colloids have been associated with adverse outcomes in adult renal transplantation.^12^ Per protocol, albumin was considered as an alternative colloid.

Reference values for normal cardiac index (CI) in children show a wide range.^13^ Patient's CI at t0 was therefore used as normal reference or baseline value. We aimed at a higher CI at t1 compared with baseline and of at least 3.5 L min^−1^ m^−2^. This value was a pragmatic choice aiming at a higher than “normal” CI for optimal perfusion of the donor kidney. The target MAP >65 mm Hg was chosen as acceptable lower margin during anesthesia as all donors had normal ABP. Both targets were evaluated for their appropriateness by visually judging the perfusion of the donor kidney at the moment of reperfusion. If needed, target CI and MAP were adjusted to higher values. Only TPTD‐CO measurements were used to guide therapy. Although the PiCCO technology allows to continuously measure CO, using pulse contour analysis, this was not used because it is considered unreliable when frequent changes in hemodynamic support occur.

### 2.6 Statistical analysis

Results for all variables are given as mean (SD) [minimum‐maximum], unless stated otherwise.

A one‐way analysis of variance (ANOVA) for repeated measurements was used to evaluate the null hypothesis that there is no difference among patients' mean values for hemodynamic physiological variables obtained at the three measuring points t0, t1 and t2. ANOVA used Greenhouse‐Geisser correction for non‐sphericity if needed. If the ANOVA revealed a difference between the values, post hoc analysis using Bonferroni multiple comparisons test was done. Student's *t*‐test for paired data was used to compare CVP measurements.

A two‐sample *t*‐test for groups with unequal variances (Welch's test) was used to compare intraoperative fluids given to our last 10 patients with those reported in two recent papers on intraoperative management in pediatric kidney transplantation.^5^, ^6^

Data were analyzed using GraphPad Prism V7.0d (GraphPad Software) and IBM SPSS Statistics V21.0. *P* < .05 was considered statistically significant.

## 3 RESULTS

Twelve patients entered the study. Table 1 shows key figures of their perioperative characteristics.

**Table 1:** Patients' perioperative characteristics

	N = 12
Gender (number of boys/girls)	8/4
Age (y)	3.2 (0.97) [1.6‐4.9]^a^
Weight (kg)	14.1 (2.4) [10.4‐18]
Body surface area (m^2^)	0.59 (0.07) [0.46‐0.69]
eGFR before transplantation (mL min^−1^ 1.73 m^−2^)	7.6 (3.5) [3.6‐15.7]
Recipient‐donor weight ratio	0.18 (0.05) [0.11‐0.28]
Duration of anesthesia (min)	294 (37) [215‐340]
Blood loss (mL kg^−1^)	8.3 (4.8) [0‐15]
Time to creatinine nadir (d after transplantation)	<2
Time to PiCCO‐catheter removal (d)	4.2 (2.8) [1‐12]
Hospital stay (d)	25.2 [15‐44]
Graft survival, 6 mo after transplantation (%)	100
eGFR, 6 mo after transplantation (mL min^−1^ 1.73m^−2^)	100.5 (27.8) [65‐155]

Abbreviation: eGFR, estimated glomerular filtration rate.

a Results are given as mean (standard deviation) [minimum‐maximum], unless stated otherwise

### 3.1 Patients' characteristics

Table 2 shows characteristics of individual patients, their eGFR before and after LDKT, and postoperative days to extubation. Preoperative echocardiography showed structural normal hearts with good systolic function in all patients. In 1 patient a pulmonary artery stenosis and a bidirectional shunt through the foramen ovale had been corrected at infancy. All donors were healthy family related adults (seven females) with a mean age of 36.2 (7.3) [24‐45] yr.

**Table 2:** Characteristics of individual patients

Patient	Age on KT	Weight	BSA	Gender	Dialysis or Pre‐emptive	Kidney disease	eGFR		Time to extubation
							before KT 6 mo after KT		
	(y)	(kg)	(m^2^)	(m/f)			(mL min^−1^ 1.73 m^−2^)		(d after KT)
1	4.2	14.6	0.62	f	P	dysplastic kidneys	6.4	70	1
2	2.9	13	0.54	m	D	urethral valves	7.2	74	4
3	2.9	18	0.69	m	P	urethral valves	3.6	95	3
4	3.5	14.6	0.62	m	P	urethral valves	6	79	2
5	3.2	14.2	0.6	m	P	dysplastic kidneys	4.7	65	3
6	2.4	13.2	0.58	m	P	urethral valves	8	103	4
7	2	10.4	0.47	m	P	dysplastic kidneys	6.7	130	5
8	1.6	10.6	0.46	f	D	ciliopathy	10.8	130	0
9	3.3	15.5	0.65	m	P	nephrotic syndrome	15.7	86	0
10	3	12	0.55	f	D	nephrotic syndrome	4.2	155	0
11	4.4	15.7	0.63	m	P	dysplastic kidneys	6	105	0
12	4.9	17.2	0.69	f	P	ciliopathy	12	114	0

Abbreviations: BSA, body surface area (Mosteller formula); D, patient on dialysis before transplant; eGFR, estimated glomerular filtration ratio; KT, kidney transplantation; m/f, male/female; mo, months; P, pre‐emptive transplantation.

### 3.2 Intraoperative hemodynamic therapy

At t0, four patients received norepinephrine 0.07 (0.04) [0.01‐0.1] mcg kg^−1^ min^−1^ and one received dobutamine (3.8 mcg kg^−1^ min^−1^). Following reperfusion of the donor kidney all recipients experienced a decrease in ABP of >20%. Patients' target MAP and CI were quickly restored using fluid loading and increasing vasopressor therapy. After reperfusion, all patients received norepinephrine (maximum dose 0.45 (0.3) [0.1‐0.9] mcg kg^−1^ min^−1^) and eight received dobutamine (maximum dose 3.4 (1.7) [1.9‐6.3] mcg kg^−1^ min^−1^).

**Figure 2:**
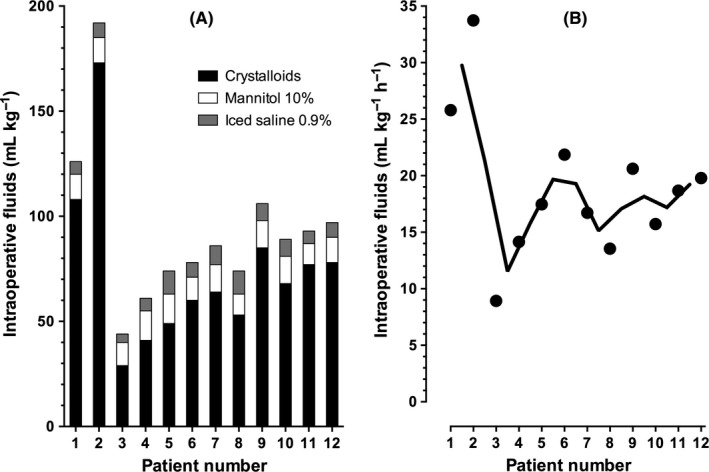
Intravenous fluids administered to the consecutive patients during surgery (n = 12). Patients are numbered consecutively as in Table 2. 2A: Total fluids expressed as mL·kg^−1^. Fluids are crystalloid (including sodium bicarbonate) and mannitol solutions plus the iced saline 0.9% used for the measurement of cardiac output with transpulmonary thermodilution. 2B: Total fluids expressed as mL·kg^−1^·h^−1^. Each black circle represents one patient. The thick solid line is a 2‐patient moving average.

Figure 2A shows the fluids administered intraoperatively. Patients received a total of 93 (37) [44‐191] mL kg^−1^ that included crystalloids, mannitol and the iced normal saline used for the CO measurements. Per protocol, of this total, 20 mL kg^−1^ were given prior to reperfusion. Fluid administration was 158 [124‐191] mL kg^−1^ in the first two patients. It decreased to 80 (18) [44‐106] mL kg^−1^ in the last 10 patients, which is 23 (8) mL kg^−1^ less than the 103 (61) mL kg^−1^ of crystalloids found by Michelet and co‐authors^5^ (95% CI = 6‐40 mL kg^−1^; *P* = .01). They also reported 16 (14) mL kg^−1^ albumin being given apart from the crystalloids. Our patients did not receive albumin.

Figure 2B shows the amount of fluids per kg body weight given hourly. Patients received 18.9 (6.4) [8.9‐33.7] mL kg^−1^ h^−1^. The first two received 29.8 [25.8‐33.7] mL kg^−1^ h^−1^, the last ten 16.7 (3.8) [8.9‐21.9] mL kg^−1^ h^−1^. The difference with the amount found by Taylor and co‐authors^6^ was not significant (95% CI = −3.0‐3.5 mL kg^−1^ h^−1^; *P* = .87).

Eight patients received blood products: 14 (8) [6‐28] mL kg^−1^. Considering crystalloids and blood products together, patients received 102 (38) [62‐207] mL kg^−1^.

### 3.3 Hemodynamic values

All hemodynamic data could be collected on the three measuring points, except for CVP. CVP was measured in 10 patients at t0 and t1.

Table 3 shows average absolute values for all hemodynamic variables, differences among the three measuring points and *P*‐values. Results of repeated measures ANOVA indicated a statistically significant time effect for HR, SVI, CI and MAP. Follow‐up comparisons indicated that HR, SVI, CI, and MAP increased between t0 and t1. There was a further increase in MAP between t1 and t2, but not for HR, SVI and CI. MAP increased with 66% (95% CI = 34%‐98%) between t0 and t2. At t2 none of the patients received norepinephrine or dobutamine. CVP values did not change between t0 and t1. Also, no statistically significant time effect for EVLWI was found.

**Table 3:** Values of hemodynamic variables obtained at three different time points: pre‐transplantation (t0), post‐reperfusion (t1) and in the intensive care unit, without hemodynamic support (t2) (n = 12)

	Absolute values			Differences between absolute values		
	(mean (SD) [minimum‐maximum])			(mean (95% confidence interval))		
	t0	t1	t2	t1‐t0	t2‐t1	t2‐t0
HR (bpm)	99 (18) [67‐125]	116 (13) [96‐135]	119 (23) [83‐162]^a^	17 (2‐32)^b^ ^1^	3 (−17‐23)^2^	20 (5‐35)^b^ ^3^
SVI (mL m^−2^)	45 (7) [32‐59]	53 (7) [38‐65]	49 (10) [29‐64]^a^	8 (3‐12)^b^ ^4^	−4 (−9‐1)^5^	4 (−3‐11)^6^
CI (L min^−1^ m^−2^)	4.4 (1.0) [2.1‐6.2]	6.0 (0.8) [4.6‐7.7]	5.7 (1.3) [2.4‐7.4]^a^	1.6 (1.0‐2.3)^b^ ^7^	−0.3 (−1.2‐0.6)^8^	1.3 (0.5‐2.1)^b^ ^9^
MAP (mm Hg)	59 (13) [46‐83]	73 (7) [62‐83]	94 (19) [62‐116]^a^	14 (6‐22)^b^ ^10^	21 (6‐36)^b^ ^11^	35 (16‐53)^b^ ^12^
CVP (mm Hg)	9.5 (3) [5‐14]	9.8 (4) [4‐17]	c	0.3 (−1.9‐2.5)	c	c
EVLWI (mL·kg^−1^)	13 (6) [8‐29]	12 (3) [8‐17]	11 (4) [6‐17]	NA	NA	NA

Note

NA means not applicable because the ANOVA for repeated measurements yielded *P* = .31 (EVLWI).

Abbreviations: CI, cardiac index; CVP, central venous pressure; EVLWI, extravascular lung water index; HR, heart rate; L, liter; MAP, mean arterial pressure; SVI, stroke volume index.

a Statistically significant effect of time (repeated measures ANOVA); *P*‐values are: .01 (HR), .007 (SVI), <.0001 (CI) and <.0001 (MAP).

b Statistically significant difference (95% confidence interval includes zero) between time points; Bonferroni adjusted *P*‐values are as follows: .022^(1)^, >.999^(2)^, .008^(3)^, .002^(4)^, .154^(5)^, .472^(6)^, <.0001^(7)^, >.999^(8)^, .003^(9)^, .001^(10)^, .007^(11)^ and .0007^(12)^.

c CVP was obtained in 10 patients at 2 times points; the difference between values for CVP was not statistically significant (Student's *t*‐test for paired data: *P* = .76).

**Figure 3:**
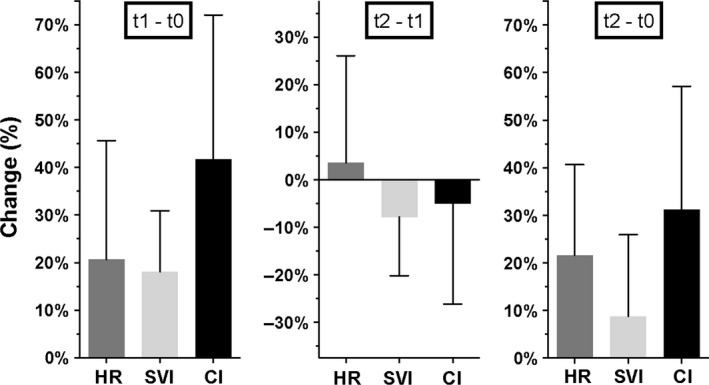
Percentage changes between three measuring points for three physiologically coupled variables: HR, heart rate; SVI, stroke volume index and CI, cardiac index. An error bar represents one standard deviation (n = 12). The three y‐axes have the same length of 75%. t0: after induction of anesthesia, before surgical incision; t1: after reperfusion of the donor kidney during a stable hemodynamic situation; t2: after cessation of hemodynamic support, at the intensive care unit

Figure 3 shows the percentage changes between the three measuring points for the physiologically coupled variables HR, SVI, and CI. Between pretransplantation (t0) and post‐transplantation measuring points (t2) CI increased with a mean of 31% (95%CI = 15%‐48%) and HR with 22% (95%CI = 9%‐34%). SVI increased between t0 and t1 with 18% (95%CI = 10%‐26%), but did not show a statistically significant change between t0 and t2.

### 3.4 Postoperative data

All donor kidneys displayed diuresis shortly after reperfusion. Early complications were drug‐induced delirium in two patients, septicemia in one and early postoperative hemorrhage in another, necessitating reoperation. In a patient with nephronophthisis (ciliopathy with medullary renal cysts) and pre‐existent liver fibrosis, excessive postoperative ascites and lymph leakage were present. Perfusion problems of the leg distal from the PiCCO catheter or complications at the site of anastomosis were not encountered. After ICU admission, vasopressor therapy could be tapered down in all patients. The PiCCO catheter was removed in ICU when inotrope and vasopressor infusions were no longer needed. Time to removal varied substantially 4.2 (2.8) [1‐12] days. In the first patients of the cohort, the catheter stayed in for 5 days after operation. This gradually decreased to 1 day in the last two patients. One patient had PiCCO monitoring up to 12 days post‐transplantation due to an increased need for sedatives leading to hemodynamic instability, excessive fluid administration, pulmonary edema, prolonged ventilation, and delirium. After this, new protocols aimed at cessation of sedation and ventilation within 24 hours after transplantation, consequently reducing the duration of hemodynamic support and monitoring.

None of the kidneys showed delayed graft failure or early graft rejection. Patient and graft survivals were both 100% at 6 months after kidney transplantation.

## 4 DISCUSSION

In this pilot study, TPTD appeared a feasible and safe technique to measure CO during LDKT with large donor‐recipient size mismatch. This is also the first report to quantify CO changes in these patients using a method that was recognized as gold standard for children.^9^, ^10^ After transplantation, cardiac index increased with 31% (95% CI = 15%‐48%). An unambiguous answer to the question whether CO‐guided hemodynamic therapy restricts intraoperative fluid administration was not found. Our results suggest that there was a trend toward less fluids given in favor of vasopressor use to achieve target ABP. Postoperative renal function was excellent.

### 4.1 Feasibility and safety

The TPTD‐CO monitor has shown its feasibility, reliability and safety in pediatric intensive care for many years.^14^ Our results confirm these findings and show that this method can be used during pediatric kidney transplantation when large hemodynamic changes are expected. Cardiac output measurements were easily performed and did not interfere with surgical activities. Concerns about the intra‐arterial catheter needed for this technique are thrombosis and ischemia of the limb distal to the catheter. These complications have been reported in neonates and infants with severe systemic circulation problems.^15^ In our patients, no such problems were encountered despite a prolonged use in some of them. This might be explained by their relatively older age and the absence of sepsis or circulatory shock. Clearly, the benefits of a CO monitor using an indwelling arterial catheter should always be weighed against its risks.

### 4.2 Physiology

Hemodynamic changes after reperfusion of the donor kidney reflect those occurring after suddenly opening a large arterio‐venous shunt as described by Guyton and Sagawa in 1961.^16^ In their model, opening this shunt resulted in a sudden reduction of the systemic vascular resistance, causing an increased CO and subsequent increase in venous return. These hemodynamic changes occur also after creating an arterio‐venous‐fistula in adults.^17^ The resultant initial decrease in ABP is counteracted by the baroreceptor reflex, increasing HR and systemic vascular resistance. When this reflex is suppressed, as during anesthesia, hypotension is likely to occur. Thus, both CO and the systemic vascular resistance must increase to maintain an adequate ABP.

Our results are concordant with this mechanism. We found a 31% increase in CI between t0 and t2, which is similar to reported values.^3^, ^4^ Furthermore, our baseline CI values correspond well with those obtained in normal children. The difference with CI values obtained by Krovetz^18^ is 0.08 (95%CI = −0.52‐0.68) L min^−1^ m^−2^. At t2, all hemodynamic support had been stopped. Therefore, CO increase between t0 and t2 can be attributed to the relatively large donor kidney, despite the pharmacological effects of anesthetics and volume loading during transplantation and in the ICU. The absence of general anesthesia at t2 obviously aided the increase in HR. The increase in MAP between t0 and t2 suggests a permanent restoration of systemic vascular resistance, aided by the absence of anesthetics at t2.

### 4.3 Hemodynamic therapy

#### 4.3.1 Pressure‐guided

Considering the described mechanism, hemodynamic therapy guided only by ABP and CVP lacks physiological grounds. Although an adequate ABP is necessary to prevent acute kidney injury, ABP values alone only provide limited information on renal perfusion and oxygenation.^19^ CVP is influenced by many factors and is known to be insufficient to predict fluid status.^7^, ^20^ Studies in adults show that delayed graft function in kidney transplantation is related to postoperative ABP but not to CVP.^21^ Moreover, higher CVP levels and fluid overload are associated with higher risks of acute kidney injury, renal replacement therapy, and mortality in critically ill children.^22^, ^23^ In a retrospective analysis, postoperative renal replacement therapy was related to high volumes of intra‐operative fluid administration related to body weight and a higher donor‐recipient weight ratio.^5^ This supports the idea that CVP‐guided fluid administration may lead to tissue edema, especially in small children.

#### 4.3.2 Flow‐guided

Optimal renal blood flow and pressure are of paramount importance to prevent hypoperfusion, delayed graft function, and subsequent loss of renal tissue. Especially in small children, it is a challenge to achieve this as both hypovolemia and excess fluids are detrimental for the renal microcirculation. It would be ideal if renal flow, pressure, and microcirculation could be estimated. Our algorithm uses second bests as CO (renal flow), MAP (renal perfusion pressure) and fluid responsiveness (volume status). With the currently available hemodynamic monitors fluid responsiveness is difficult to assess in children.^20^ CO measurements also cannot directly estimate fluid responsiveness, but are able to exhibit the effects of fluid loading and vasopressor therapy on CO.

#### 4.3.3 Norepinephrine

In addition to fluids, we administered dobutamine and norepinephrine to support CO and ABP. Using dobutamine did not result in tachycardia. Norepinephrine was administered to support ABP by counteracting the depressant effects on systemic vascular resistance of (a) anesthetics and anti‐hypertensive drugs, (b) opening the arterial anastomosis and (c) cytokines released after reperfusion of the (ischemic) donor kidney. After an ischemic period, autoregulatory mechanisms in the donor kidney might be disturbed and pharmacological support may be needed to guarantee an adequate perfusion pressure. In the past, concerns existed on the vasoconstrictive aspects of norepinephrine and its possible negative effects on visceral organ perfusion. Recent studies show otherwise: in a vasodilated state norepinephrine can even improve renal blood flow. It is currently the drug of choice to prevent hypotension in septic shock without increasing the incidence of acute kidney injury.^24^ In our patients, maximum doses were reached shortly after reperfusion and were only needed for a few hours and tapered down in the ICU after reduction in the level of sedation. Administration stopped before PiCCO‐catheter removal. Our results suggest that CO‐guided administration of norepinephrine can be used to achieve target ABP without negative effects on renal outcome.

#### 4.3.4 Fluids

As CVP and EVLWI did not increase despite fluid loading, we suggest that our hemodynamic strategy did not cause fluid overload. Prolonged time to tracheal extubation can be a sign of pulmonary edema and fluid overload. In our study, the moment of extubation depended on many variables, including potential signs of fluid overload and the sedation protocol. The latter was optimized during the study, which gradually led to shorter time to extubation. Table 2 shows that the last five patients could be extubated on the day of transplantation.

To assess whether our strategy led to less intraoperative fluids, there were only two recent studies to compare our results with, although the cohorts differ in age, weight, and the allograft source.^5^, ^6^ Compared with Michelet's study,^5^ we gave 25% less crystalloids to the last 10 patients and no albumin. Our fluid amounts were similar to those found in Taylors study,^6^ expressed as mL kg^−1^ h^−1^. However, this disregards the fact that all our patients weighed <20 kg vs 36.2 kg for Taylor's average patient. Our last 10 patients received 4.9 (1.2) [2.8‐6.3] times their basic fluid requirement calculated with the 4‐2‐1‐rule vs 8.1 times for Taylor's average patient. Obviously, prudence is required in judging the validity of this calculation for the average patient.

### 4.4 Alternative CO‐monitoring

Although the TPTD‐CO monitor uses a validated and reliable technique, it is invasive and requires experience. Echocardiography is less invasive and can supply the physician with useful information but has several limitations, such as technical problems during surgical procedures, operator dependency, and variability. Non‐ or minimally invasive CO monitors are easily applicable but also require experience while their reliability is questionable in young children and at best may be used as a trend monitor.^25^ Apparently, there is increasing interest in advanced hemodynamic monitoring in pediatric kidney transplantation. Especially, esophageal doppler monitoring^26^ seems to gain popularity in this field. Although it is still difficult to show an unambiguous impact on outcome, monitoring the effect of fluids, and vasopressor support on CO makes sense as a cornerstone in hemodynamic management. Above all, the usefulness of any CO‐monitor depends on the operators' experience and knowledge of the technical reliability issues.

### 4.5 Limitations

Our study did not use a control group without CO monitoring. The first 12 patients in our program served as a pilot cohort to test the feasibility and safety of our hemodynamic monitoring and therapy plan. As we considered CO‐guided hemodynamic support as best clinical practice, we omitted a control group. Our cohort was small but consistent in age, weight, type of donor and short cold ischemia times. This consistency and the uniform hemodynamic protocol make our findings rather robust. Other studies show considerable variation in recipients' age and weight, donor characteristics and hemodynamic therapy, which makes comparisons difficult. Nevertheless, the small number of patients should let us refrain from very broad conclusions on safety and risk‐benefit ratio.

In conclusion, a kidney transplantation with large donor‐recipient size mismatch implies clinically significant hemodynamic changes, illustrated by a 31% increased CO. The TPTD‐technique proved to be feasible and safe in this cohort. The results suggest that optimizing hemodynamics using a CO‐guided hemodynamic therapy algorithm to titrate inotropes, vasopressors, and fluids may reduce intra‐operative fluid administration compared with a CVP‐guided approach, thus possibly preventing tissue edema. As this is a small cohort, further research is desirable focusing on the most efficient algorithm and outcome effects of CO‐guided fluid therapy in these patients.

## CONFLICT OF INTEREST

The authors report no conflict of interest.

## ETHICAL APPROVAL

The Regional Committee for Medical and Health Research Ethics (Nijmegen, The Netherlands) ethically approved this study. The Committee waived the requirement for written informed consent. Parents were informed of the study and given the opportunity to refuse the use of their child's data.
